# Deep Learning Neural Network Prediction Method Improves Proteome Profiling of Vascular Sap of Grapevines during Pierce’s Disease Development

**DOI:** 10.3390/biology9090261

**Published:** 2020-09-01

**Authors:** Cíntia Helena Duarte Sagawa, Paulo A. Zaini, Renata de A. B. Assis, Houston Saxe, Michelle Salemi, Aaron Jacobson, Phillip A. Wilmarth, Brett S. Phinney, Abhaya M. Dandekar

**Affiliations:** 1 Department of Plant Sciences, University of California, Davis, 1 Shields Ave, CA 95616, USA; chdsagawa@ucdavis.edu (C.H.D.S.); pazaini@ucdavis.edu (P.A.Z.); redab@ucdavis.edu (R.d.A.B.A.); hsaxe@ucdavis.edu (H.S.); ajacobson@ucdavis.edu (A.J.); 2 Departamento de Ciências Biológicas, Instituto de Ciências Exatas e Biológicas, Núcleo de Pesquisas em Ciências Biológicas, Universidade Federal de Ouro Preto, 122-Bauxita, Ouro Preto-MG 35400-000, Brazil; 3 Proteomics Core Facility, University of California, Davis, 1 Shields Ave, CA 95616, USA; msalemi@ucdavis.edu (M.S.); bsphinney@ucdavis.edu (B.S.P.); 4 Proteomics Shared Resource, Oregon Health and Science University, Medical Research Building, 3252 SW Research Drive, Portland, OR 97239, USA; wilmarth@ohsu.edu

**Keywords:** predicted spectral library, quantitative proteomics, Prosit, apoplast, xylem sap, grapevine, Pierce’s Disease, secretome

## Abstract

Plant secretome studies highlight the importance of vascular plant defense proteins against pathogens. Studies on Pierce’s disease of grapevines caused by the xylem-limited bacterium *Xylella fastidiosa* (*Xf*) have detected proteins and pathways associated with its pathobiology. Despite the biological importance of the secreted proteins in the extracellular space to plant survival and development, proteome studies are scarce due to methodological challenges. Prosit, a deep learning neural network prediction method is a powerful tool for improving proteome profiling by data-independent acquisition (DIA). We explored the potential of Prosit’s in silico spectral library predictions to improve DIA proteomic analysis of vascular leaf sap from grapevines with Pierce’s disease. The combination of DIA and Prosit-predicted libraries increased the total number of identified grapevine proteins from 145 to 360 and *Xf* proteins from 18 to 90 compared to gas-phase fractionation (GPF) libraries. The new proteins increased the range of molecular weights, assisted in the identification of more exclusive peptides per protein, and increased identification of low-abundance proteins. These improvements allowed identification of new functional pathways associated with cellular responses to oxidative stress, to be investigated further.

## 1. Introduction

The vascular system is essential for information exchange and resource allocation throughout the plant, from roots to aerial tissues. It is composed of two vascular tissue types: phloem and xylem. The phloem sap contains photoassimilates and other macromolecules that move throughout the plant from areas of synthesis or excess (source) to areas of use (sink) and storage [1]. The xylem sap transports water and nutrients from roots to aerial tissues, driven by a difference in water potential due to transpiration [2]. Xylem sap can also contain a wide range of proteins involved in growth regulation, protection against environmental stress, and plant defense against pathogens [3]. These biological processes depend on vesicular trafficking of proteins to the extracellular space, which can follow either conventional or unconventional secretion routes in plant cells. Conventional secretion requires N-terminal signal peptides or other recognition signals to direct them to the endomembrane system pathway, while proteins that follow the unconventional secretion route lack these signals [4]. Proteins that follow unconventional secretion can allow plants to respond to a wider range of extracellular stresses and stimuli, facilitating defense responses under stress [4,5]. Despite the biological importance of secreted proteins in the extracellular space to plant survival and development, proteome studies are scarce because of technological challenges.

Vascular sap studies have advanced our understanding of plant responses to vascular plant diseases [6]. The Gram-negative gammaproteobacterium *Xylella fastidiosa* (*Xf*) is a xylem-limited pathogen that colonizes several economically important crops worldwide causing diseases such as Pierce’s disease (PD) in grapevines [7], citrus variegated chlorosis (CVC) [8], and most recently olive quick decline syndrome (OQDS) in Europe [9]. Because of its significant economic impact on citrus production in Brazil, *Xf* was the first plant pathogen to have its genome sequence determined [10]. The genomic landscape provided an initial description of potential virulence factors and revealed the absence of a type III secretion system commonly employed by plant pathogens to deliver virulence effectors inside plant cells. Subsequent molecular and cellular studies proposed that the mechanism of disease symptoms would be associated with biofilm formation and xylem blockage triggering the observed disease symptoms [11,12,13,14,15]. Additionally, genomics and proteomics showed the importance of virulence factors secreted by the type II secretion system and outer membrane vesicles for symptom development [16,17,18,19,20]. These studies highlighted the molecular complexity of the plant-pathogen interaction that takes place in the vascular system [21,22,23,24,25,26] (Table 1).

The importance of proteins in the plant response to *Xf* was detailed in several proteome studies comparing infected and uninfected grapevine stems [23] and the infection responses of different cultivars [25]. These studies identified more than 200 proteins associated with disease resistance, energy metabolism, protein processing and degradation, biosynthesis, stress-related functions, cell wall biogenesis, signal transduction, and ROS detoxification among others. A most recent study of sap bleeding of infected grapevines incorporated structural data into the proteomic data analysis to enhance identification of functionally relevant protein candidates that would be undetectable from simple amino acid sequence alignments [26]. These studies have greatly enhanced the understanding of xylem sap physiology; however, they were restricted to more abundant proteins which are only a small fraction of xylem sap complexity.

Peptide (and protein) detection can be improved by alternative mass spectrometer data acquisition schemes like data-independent acquisition (DIA) which is based on acquisition of fragment-ion information for all precursor ions until the desired mass range has been covered [27]. Although it improves peptide detection with greater reproducibility, the need for accurate measured or predictive models for fragment ion intensities limits its potential. DIA analysis often uses peptide physiochemical properties stored in spectral libraries or chromatogram libraries. Pooled samples can be used in gas-phase fractionation (GPF) to improve detection rates by breaking down the acquision in specific fractions to generate DIA-only chromatogram libraries to facilitate peptide detection in single injection DIA samples [28]. Peptide physiochemical properties can include information on peptide retention time, product ion *m*/*z*, product ion intensity, and ion mobility among others [29]. Peptide properties can be obtained by experimental or predictive methods. Predictive models of peptide LC-MS/MS properties based on deep learning neural network methods have now been developed [30,31,32,33,34,35]. One of these methods is called Prosit [31]. It predicts chromatographic retention time and fragment ion intensity based on sequences of synthetic peptides and tandem mass spectra generated within the ProteomeTools project [36] which can exceed the quality of experimental data on animal and bacterial proteomes [31,37]. Here we demonstrate the improved performance of integrating Prosit into a DIA workflow. By reanalyzing our DIA data on the vascular leaf sap of healthy and *Xf*-infected grapevines by using Prosit predictions instead of GPF, we significantly increased the number of identified proteins involved in this plant-pathogen interface generating in-silico spectral libraries for DIA analysis of grapevines (*Vitis vinifera*) and *X. fastidiosa* that can be incorporated into future proteome studies.

## 2. Material and Methods

### 2.1. Plant Material and X. fastidiosa Inoculation

Clonal grapevine plants (*Vitis vinifera* L. cv. ‘Thompson Seedless’) were generated from cuttings using green canes from the current season’s growth. Each cutting was ~six inches long and contained two nodes, with a petiole originating from the top node that supported ~one square inch of leaf area to maintain minimal photosynthesis during rooting. These prepared cuttings were placed into an EZ-Clone aeroponic cloning system that circulates water purified by reverse osmosis. Roots begin to self-generate after two weeks, and the rooted cuttings were potted after three-weeks and grown in a greenhouse. New plant growth was trained to a single cane by removing any lateral shoots that emerged and topped at the height of one meter. Additional lateral shoots were removed as they emerged during the experiment. After ten weeks, the grapevines were infected at eight to 12 cm above soil level by punching with a needle gauge to inoculate 20 μL of cultured *Xylella fastidiosa* Temecula1 (*Xf*; ATCC 700964) cells into the stem as described by Nascimento et al. (2016) [16]. The bacterial culture was grown on PD3 medium at 2 × 10^8^ cells/mL with aeration (120 rpm) at 28 °C. After inoculation, experimental and non-inoculated control (healthy) plants were placed in the greenhouse in a randomized block design and monitored for 12 weeks post inoculation until leaf symptoms developed.

### 2.2. Vascular Sap Extraction and X. fastidiosa Quantification

Vascular leaf sap was collected from ten leaves above the inoculation point using a pressure chamber (Soil Moisture Equipment Corp., Santa Barbra, CA, USA). Pressure was applied to each leaf blade and the sap was collected from the end of the petiole. The leaf blade was placed inside the pressurized chamber leaving only the cut surface of the petiole exposed to release the vascular content, which was collected using a micropipette and stored in a tube on ice during harvest. Pools of sap from ~ ten leaves from above the inoculation point on one plant comprised a sample (500 to 1000 μL). Before processing for proteomics analysis, 25 μL was reserved from each sample for extraction of DNA with the MasterPure™ Complete DNA and RNA Purification kit (Epicentre Technologies, Madison, WI, USA) and bacterial cell count was measured using qPCR (TaqMan™, Thermo Fisher Scientific, Waltham, MA, USA). The primers used were HL5 and HL6 described by Francis et al. (2006) [38]. A standard curve was used based on a known serial dilution of *Xf* cells measured by OD_600_.

### 2.3. Protein Digestion of Vascular Leaf Sap

Up to one mL vascular leaf sap was collected from each plant (pooled from ten leaves) and a total of three plants per group (healthy or diseased) was used. Samples were centrifuged at 5000 rcf for five min at 4 °C. The supernatant containing the vascular leaf sap was transferred to a new tube. Total protein content was quantified by Qubit™ Protein Assay Kit (Thermo Fisher Scientific). Sap containing 100 ug protein was freeze-dried and resuspended in 5% SDS and 50 mM triethylammonium bicarbonate (TEAB) at pH 7.55 to a concentration of 0.5 μg/μL. Digestions with trypsin followed the S-Trap™ Micro Spin Column Digestion Protocol (Protifi) with few modifications. Initially, 10 mM dithiothreitol (DTT) was added and incubated at 50 °C for 10 min and rested at room temperature for 10 min. Next, 5 mM iodoacetamide (IAA) was added and incubated at room temperature for 30 min in the dark. The samples were acidified with 12% phosphoric acid followed by addition of 2.348 mL freshly made S-trap (St) buffer (90% methanol, 100 mM TEAB, pH 7.1) and mixed immediately by inversion. The entire acidified lysate/St-buffer mix was transferred to the S-trap spin column (650 μL at a time) and centrifuged at 3000 rcf for one minute or until all the solution passed through the column. Columns were washed with 400 μL S-trap buffer and centrifuged at 4000 rcf until dry. Columns were transferred to a clean elution tube. Trypsin enzyme digest buffer was carefully added (1:25 enzyme: total protein in 121 μL 50 mM TEAB, pH 8.0) to the column and followed by incubation at 37 °C overnight. After the first hour, the trypsin digestion step was repeated. Peptide elution steps included 80 μL of 50 mM TEAB (pH 8.0) followed by centrifugation at 1000 rcf for one minute, 80 μL of 0.5% formic acid followed by centrifugation at 1000 rcf for one minute, then 80 μL of the solution containing 50% acetonitrile and 0.5% formic acid followed by centrifugation at 4000 rcf for one minute. The final pooled elution was dried down in a speed-vacuum. Peptides were resuspended in 0.1% TFA 2% ACN and quantified using Pierce™ Quantitative Fluorometric Peptide Assay (Thermo Fisher Scientific). Equal portions of all samples were mixed together to make a reference sample to be run multiple times for chromatogram library runs.

### 2.4. Liquid Chromatography Tandem Mass Spectrometry

All proteomics methods were performed at the UC Davis Proteomics Core Facility. Peptides were trapped on a Thermo PepMap trap and separated on an Easy-spray 100 μm × 25 cm C18 column using a Dionex Ultimate 3000 nUPLC at 200 nL/min. Solvent A = 0.1% formic acid and Solvent B = 100% acetonitrile with 0.1% formic acid. Gradient conditions were 2% B to 50% B over 60 min, followed by 50% to 99% B in six min held for three min, then 99% B to 2% B in two min for a total run time of 90 min using a Thermo Scientific Fusion Lumos mass spectrometer running in data independent acquisition (DIA) mode.

### 2.5. Chromatogram Library Creation

Six gas-phase fractionated (GPF) chromatogram library injections were made using staggered 4 Da isolation windows. They were GPF1: 400–500 *m*/*z*; GPF2: 500–600 *m*/*z*; GPF3: 600–700 *m*/*z*; GPF4: 700–800 *m*/*z*; GPF5: 800–900 *m*/*z*; GPF6: 900–1000 *m*/*z*. Mass spectra were acquired using a collision energy of 35, resolution of 30 K, maximum inject time of 54 ms, and a AGC target of 50 K.

### 2.6. Analytic Samples, Data Analysis and Raw Data Processing

Each individual sample was run in DIA mode using the same settings as the chromatogram library runs except using staggered isolation windows of 12 Da in the *m*/*z* range 400–1000 *m*/*z*. DIA data were analyzed using Scaffold DIA v.2.0.0 (Proteome Software, Portland, OR, USA). Raw data files were converted to mzML format using ProteoWizard v.3.0.11748 [39].

### 2.7. Spectral Library Search

**Figure 1:**
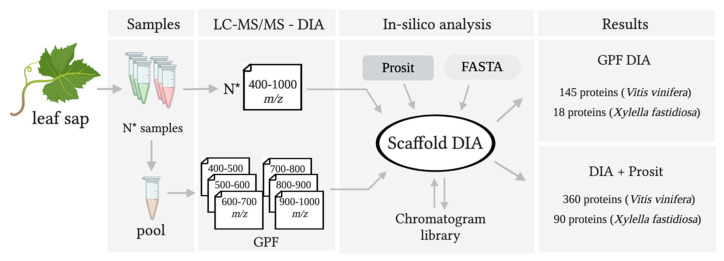
Quantification of peptides with chromatogram libraries workflow from grapevine leaf sap with Pierce’s disease. The chromatogram library generation was based on Searle et al. (2018). Created with BioRender.com.

The Reference Spectral Library was created by EncyclopeDIA v.0.9.2 (Figure 1). Chromatogram library samples were searched individually against Prosit-predicted databases created using the Prosit online server (https://www.proteomicsdb.org/prosit/) and converted for ScaffoldDIA using the EncyclopeDIA tools [28]. The input for the Prosit prediction consisted of UniProt proteome UP000009183 (*Vitis vinifera*, Grape), UniProt proteome UP000000812 (*Xylella fastidiosa*), and 114 common laboratory contaminants (https://www.thegpm.org/crap/) with a peptide mass tolerance and a fragment mass tolerance of 10.0 ppm. Variable modifications considered were oxidation of methionine and static modifications were carbamidomethyl of cysteine. The digestion enzyme was assumed to be trypsin with a maximum of one missed cleavage site allowed. Only peptides with 2+ or 3+ charges and length range between 6 and 30 (inclusive) were considered. Peptides identified in each search were filtered by Percolator (3.01.nightly-13-655e4c7-dirty) [40,41,42] to achieve a maximum false discovery rate (FDR) of 0.01. Individual search results were combined, and peptides were again filtered to an FDR threshold of 0.01 for inclusion in the reference library.

### 2.8. Quantification and Criteria for Protein Identification

Peptide quantification was performed by EncyclopeDIA v.0.9.2. For each peptide, the five highest-quality fragment ions were selected. Proteins that contained similar peptides and could not be differentiated based on MS/MS analysis were grouped to satisfy the principle of parsimony. Only proteins with a minimum of two identified peptides were considered and filtered by a protein FDR threshold of 1.0%. Raw data and ScaffoldDIA results were deposited at the proteome repositories MassIVE (massive.ucsd.edu) and at ProteomeExchange (http://www.proteomexchange.org/) accession numbers #MSV000085942 and #PXD020876. Differential expression testing was done in Jupyter notebooks (https://jupyter.org/) using an R kernel and the Bioconductor package edgeR [43,44] (Supplemental html files).

### 2.9. Functional Enrichment Analysis

The functional analysis of vascular leaf sap proteome of grapevines was performed with Metascape [45] using the express analysis settings. The IDs of *Vitis vinifera* (VIT) of increased and decreased proteins in diseased samples were converted into the corresponding *Arabidopsis* homolog protein IDs and analyzed independently. The *Arabidopsis* homologs were identified in TAIR using Protein Basic Local Alignment Search Tool (BLASTP). Metascape identified pathways and GO biological process enrichment analysis were defined by the Kyoto Encyclopedia of Genes and Genomes (KEGG). *p*-value was adjusted by the method of Benjamin-Hochberg to control the FDR [45].

## 3. Results

### 3.1. Creating a DIA Library and Improving the Data Mining of Vascular Sap Proteome Data

The proteome of vascular leaf sap from healthy grapevines were compared to those developing PD symptoms because of *X. fastidiosa* (*Xf*) infection. Infection was confirmed by qPCR that quantified a high number of bacterial cells (1.5 × 10^9^ cells/mL) present in the diseased samples (Table S1). Comparative proteomics was employed to investigate molecular aspects of Pierce’s disease as described (Figure 1), showing the conventional DIA pipeline with and without Prosit.

**Figure 2:**
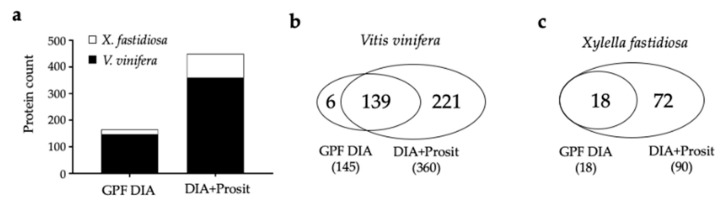
Vascular leaf sap proteomic analysis of *Vitis vinifera* and *Xylella fastidiosa*: (**a**) total proteins identified by data-independent acquisition (GPF DIA) and DIA+Prosit; (**b**) Venn diagram of the number of proteins identified by each method for *V. vinifera*; and (**c**) for *X. fastidiosa*.

The proteome results obtained with both pipelines were compared (Figure 2). GPF DIA analysis identified 145 and 18 proteins for *V. vinifera* (VIT) (Table S2) and *Xf* (Table S3), respectively. After integrating Prosit-generated libraries into the database search pipeline, the number of proteins increased by 148% for VIT and 400% for *Xf*, to a final total of 360 and 90 proteins, respectively (Tables S4 and S5). Just six VIT proteins were identified exclusively by GPF DIA (without Prosit) and 221 only by integrating Prosit (DIA+Prosit), with 139 detected in both approaches for VIT (Figure 2b, Table S6). Among the six VIT proteins identified only by GPF DIA were four peroxidases (VIT_01s0010g01950, VIT_01s0010g01960, VIT_01s0010g02000, VIT_01s0010g02010), one is an uncharacterized protein with serine-type endopeptidase activity (VIT_16s0098g01160), and the last is a glyco-hydro 18 domain-containing protein (VIT_16s0050g02220). The proteins detected exclusively by Prosit were associated with many more molecular functions, including cell adhesion molecules, scaffold/adaptors proteins, chaperones, translational proteins, transporters, and nucleic acid-binding proteins. Eighteen *Xf* bacterial proteins were identified by both methods; however, DIA+Prosit detected an additional 72 proteins that were not present in the GPF DIA data (Table S7).

**Figure 3:**
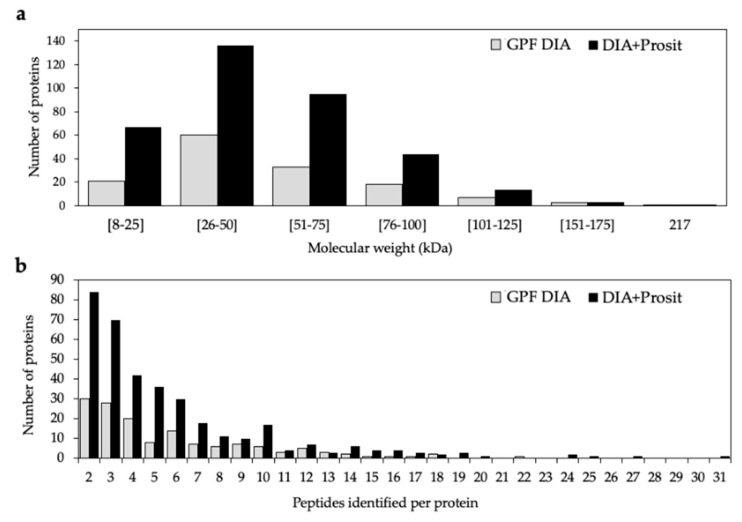
Distribution of the total number of *V. vinifera* proteins identified by GPF DIA and DIA+Prosit by (**a**) molecular weight (kDa) ranging from eight to 217 kDa. (**b**) Identified peptides varying from two to 31 peptides per protein. Predicted proteins with only one peptide were discarded.

**Figure 4:**
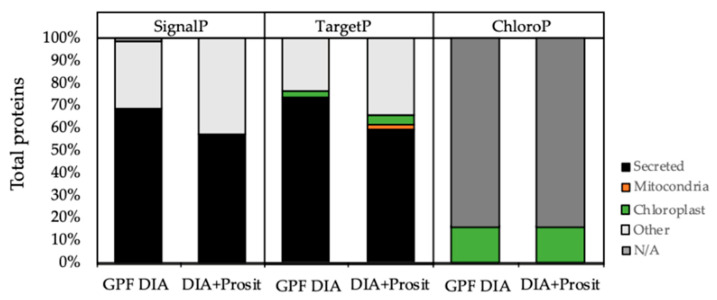
Subcellular localization prediction analysis and comparisons between GPF DIA and DIA+Prosit data using SignalP, TargetP, and ChloroP servers. More than 50% of the total proteins identified were predicted to have a signal peptide, according to SignalP and TargetP-SP. TargetP output revealed <3% of total proteins containing a mitochondrial targeting peptide (mTP) and <5% of proteins containing a chloroplast transit peptide (cTP). ChloroP predicted 16% of the collected vascular sap proteins would target the chloroplast by both methods. GPF DIA considered 145 proteins and DIA+Prosit, 360 proteins for *V. vinifera*.

The application of Prosit to our data substantially increased the number of proteins with a molecular weight below 100 kDa. The range of molecular weight varied from 12 kDa to 217 kDa in GPF DIA data and 8 kDa to 217 kDa in DIA+Prosit data. This slightly wider molecular weight range is better appreciated with a breakdown of proteins identified by both methods by molecular weight and the number of mapped peptides (Figure 3a), revealing the superior performance of the pipeline with Prosit. The smallest proteins detected by GPF DIA are 12 kDa AAI domain-containing proteins (VIT_02s0236g00020 and VIT_02s0236g00030), both increased in diseased plants. The maximum number of peptides identified per protein was 22 for GPF DIA, and 31 for DIA+Prosit. Most proteins identified after Prosit integration showed two to ten peptides per protein (Figure 3b). In DIA+Prosit data, the 8 kDa BBE domain-containing protein (VIT_10s0003g05430) with a signal peptide targeting mitochondria (mTP) according to TargetP-2.0 Sever (http://www.cbs.dtu.dk/services/TargetP/) (Figure 4) was detected. Both AAI domain-containing proteins detected by GPF DIA were also present with DIA+Prosit, along with a third AAI domain-containing protein (VIT_16s0013g00070).

The analyzed material was an enriched vascular leaf sap; thus, we determined the proportions of proteins predicted to be secreted (Figure 4). The percentage of secreted proteins with a predicted signal peptide within the total proteins detected by GPF DIA was 68% (99/145), and by DIA+Prosit was 57% (205/360), according to SignalP-5.0 Server (http://www.cbs.dtu.dk/services/SignalP/). By using TargetP to analyze the same data sets, we found similar results: 72% and 59% for GPF DIA and DIA+Prosit, respectively. The remaining were classified as non-secretory targeting the mitochondria (1–2%), chloroplast (3–4%), or other (23–34%). By performing the same analysis using the prediction tool ChloroP 1.1 Server (http://www.cbs.dtu.dk/services/ChloroP/), 16% of the proteins in both data sets would target chloroplasts; therefore, their presence in the xylem sap possibly reflects some degree of contamination of the samples with cellular contents during vacuum-assisted sap extraction or alternatively products of natural cellular and organellar degradation.

### 3.2. Plant Secreted Proteins in Response to Pierce’s Disease

**Figure 5:**
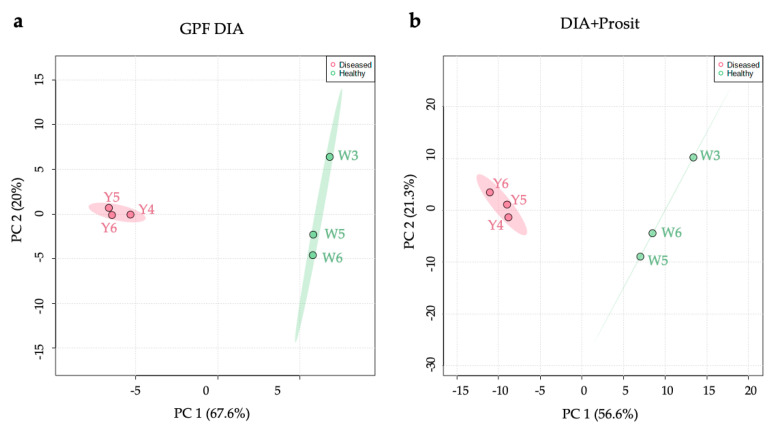
Principal component analysis (PCA) score plots of PC1 and PC2 and explained variances are shown. There was clear separation between diseased and healthy samples for *V. vinifera* using either (**a**) GPF DIA or (**b**) DIA+Prosit. W3, W5, and W6 are biological replicates of healthy plants, while Y4, Y5, and Y6 are diseased plants based on log_10_ of the exclusive intensity of peptides.

We used MetaboAnalyst 4.0 (https://www.metaboanalyst.ca) to visualize both proteome data sets and examined the variation between groups and samples [46]. The variability was examined with unsupervised principal component analysis (PCA) using the exclusive intensity of the (unique) peptides. The PCAs showed distinct separation between groups in both data sets, GPF DIA and DIA+Prosit (Figure 5). While the effects of *Xf* infection made sample clustering strikingly clear, we cannot exclude the possibility that Prosit would be decisive with more attenuated differences. Healthy and diseased groups showed 67.6% variation in PC1 for GPF DIA (Figure 5a) and 56.6% variation in PC1 for DIA+Prosit (Figure 5b). These results expose the compelling effect of *Xf* infection on the vascular leaf sap proteome. The variation among samples explained by PC2 was 20% for GPF DIA. Prosit slightly increased the variation among samples to 21.3%, explained by PC2. The protein levels in healthy and diseased group samples were distinct, independent of the method (Figure S1).

**Figure 6:**
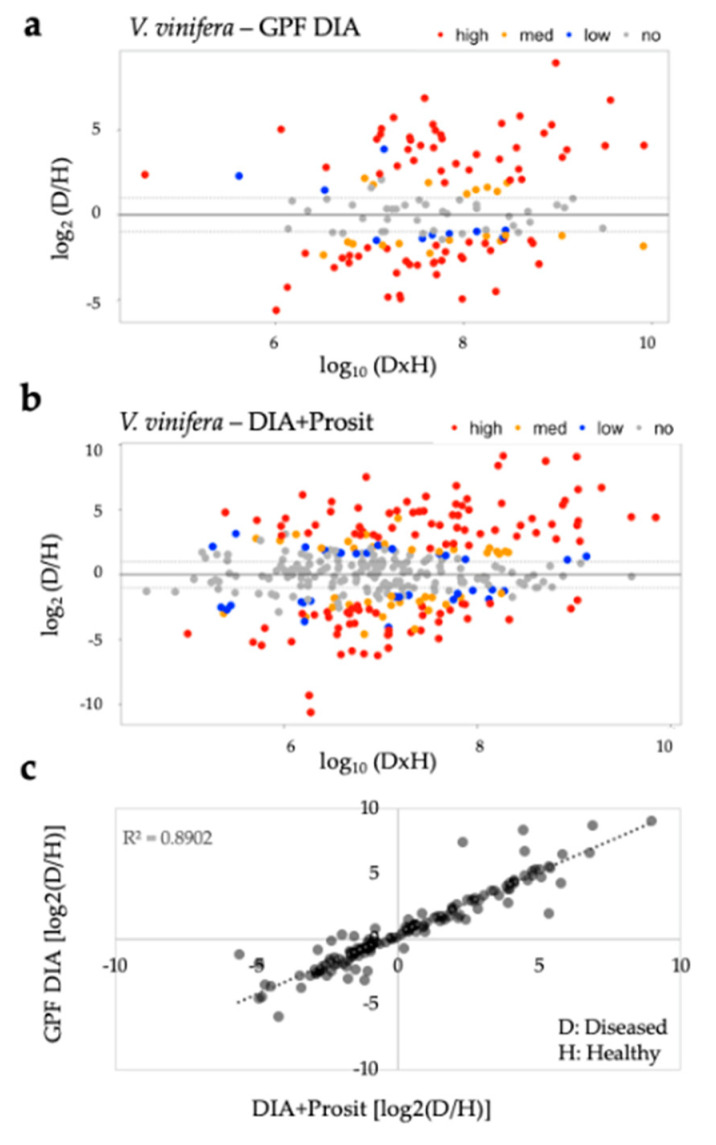
Overview of the plant response to *Xf* in diseased samples in both data sets. Analysis of ratio-intensity plots displaying the log_2_ fold-change ratio of diseased (D) over healthy (H) plants for each protein as a function of the abundance by log_10_ (DxH) product intensities, colored by FDR confidence levels: (**a**) 145 proteins identified using GPF DIA and (**b**) 357 proteins identified by DIA+Prosit; (**c**) correlation between the ratios obtained from both analyses from the proteins detected in both analysis (139) with R^2^ = 0.8902 show that the incorporation of Prosit provided similar results but with higher quality and expanded detection. D: diseased and H: healthy plants. FDR confidence levels (high: FDR ≤ 0.01; med: FDR ≤ 0.05; low: FDR ≤ 0.1; no: FDR > 0.1).

To further analyze the differences between methods, we analyzed the ratio-intensity of healthy and diseased groups and compared them to the protein abundance in both proteome data sets. The fold change of protein detection between diseased and healthy plants presented similar results for GPF DIA and DIA+Prosit data (Figure 6a–b). However, implementation of Prosit increased detection of proteins that were in low abundance, as shown by the *x*-axis in Figure 6a–b. The correlation of results of the common 139 proteins obtained by both methods was significant and had an R^2^ of 0.8902 (Figure 6c), showing that the increase of protein prediction power by Prosit correlates well with the observed data without introducing bias in differential expression.

**Figure 7:**
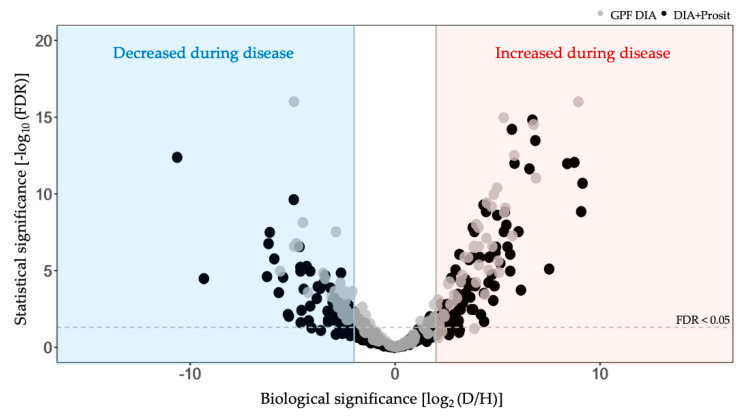
Proteome response of *V. vinifera* to *Xf* infection. Volcano plot analysis of vascular leaf sap obtained from diseased (D) and healthy (H) plants data identified by GPF DIA (145 proteins) and DIA+Prosit (353 proteins) overlapped. Proteins identified by GPF DIA are represented with grey dots and those identified by DIA+Prosit, with black dots. FDR calculated by Benjamin-Hochberg smaller than or equal to 0.05 were considered significant.

To visualize proteins that either increased or decreased significantly in the diseased group, we examined volcano plots of both data sets (Figure 7). Comparison of the log_2_-fold change of the data sets and their adjusted *p*-values by false discovery rates (FDR) show a similar profile; however, the integration of Prosit identified of additional proteins with differential abundance in the diseased group. GPF DIA identified 48 proteins increased and 47 decreased with FDR ≤ 0.05, while in DIA+Prosit, it was 84 increased and 65 decreased proteins. Seven low abundance proteins (VIT_01s0026g02070, VIT_03s0091g00130, VIT_04s0008g06040, VIT_14s0006g03230, VIT_14s0068g00680, VIT_18s0001g00510, VIT_18s0122g00960) were removed from DIA+Prosit data set due to high number of missing values among the samples. The three most-increased proteins identified by DIA+Prosit were cupredoxin superfamily protein (VIT_18s0001g11180, log_2_ FC = 9.16), beta-1,3-glucanase 3 (VIT_08s0007g06060 -PR-2 family of pathogenesis-related proteins, log_2_ FC = 9.09), and chitinase A (VIT_16s0050g02230, log_2_ FC = 8.76). The most-decreased proteins were plant invertase/pectin methylesterase inhibitor superfamily (VIT_07s0005g00720, log_2_ FC = −10.62), glyco-hydro 18 domain-containing protein (VIT_06s0004g03840, log_2_ FC = −9.31), and glyoxalase I homolog (VIT_10s0116g01660, log_2_ FC = −6.23).

**Figure 8:**
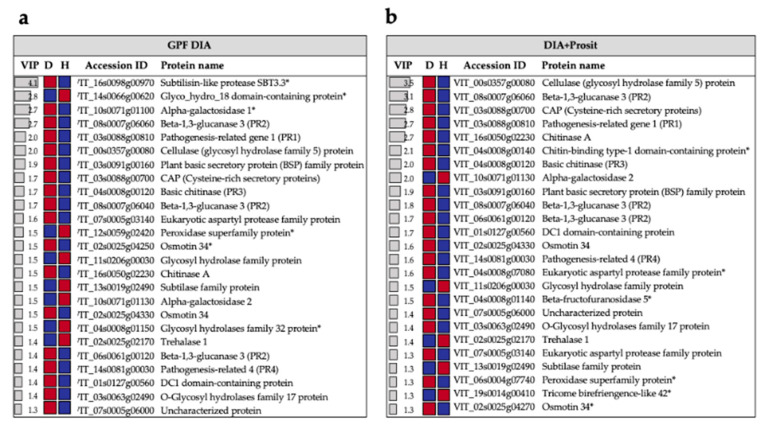
Top 25 *V. vinifera* proteins contributing to the variance between groups observed by PLS-DA. The plot shows the variable importance in projection (VIP) scores, and the colored boxes indicate the relative intensity of the corresponding protein in diseased (D) and healthy (H) plants detected by (**a**) GPF DIA and (**b**) DIA+Prosit. Red represents detection of high and blue, of low exclusive intensity. Proteins marked with an asterisk (*) are among the top 25 regardless of method. The common 139 proteins detected using both methods were used.

For a balanced comparison between methods, we used partial least squares-discriminant analysis (PLS-DA) of the 139 proteins that were detected by both methods (Figure S2). The VIP score (a metric that identifies which variables are most responsible for the differences between the classes in the analysis) of the top markers was greater in GPF DIA than in DIA+Prosit (Figure 8). Among the top 25 proteins contributing to variations between the two sample groups, we highlight the pathogenesis-related proteins (PR1, PR2, PR3, PR4) that increased in diseased plants independent of method. Only six proteins among the top 25 in GPF DIA were not in DIA+Prosit, and six proteins were in DIA+Prosit and not GPF DIA (Figure 8).

### 3.3. Enriched Biological Processes in Grapevine Vascular Leaf Sap

**Figure 9:**
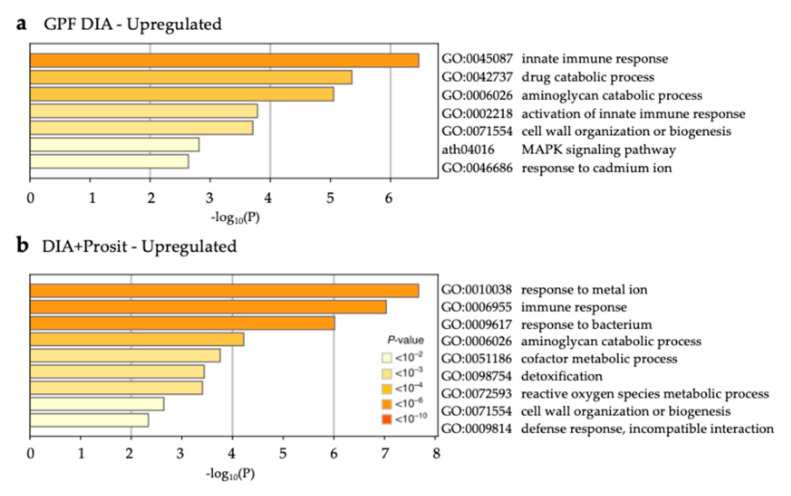
Increased GO biological processes and KEGG pathways during *Xf* infection in *V. vinifera*. Non-redundant enriched ontology clusters of significantly expressed proteins increased during *Xf* infection (*p* < 0.05) in the (**a**) GPF DIA and (**b**) DIA+Prosit data sets. DIA+Prosit identified more pathways that were likely involved with plant response to bacteria.

**Figure 10:**
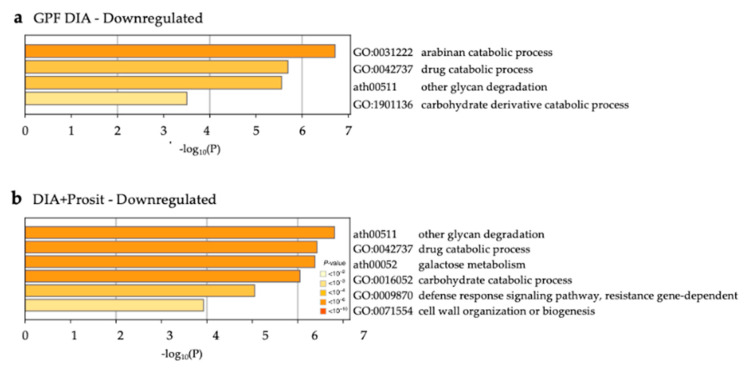
Decreased GO biological processes and KEGG pathways during *Xf* infection in *V. vinifera*. Non-redundant enriched ontology clusters of significantly expressed proteins decreased during *Xf* infection (*p* < 0.05) in (**a**) GPF DIA and (**b**) DIA+Prosit data sets. Similarly to Figure 9, DIA+Prosit identified more pathways likely involved with plant response to infection.

Representation of known enzyme pathways or protein complexes in the vascular leaf sap proteome assists in functional characterization of the plant response to infection and virulence strategies by the pathogen. Prosit identified more classes of proteins, pathways, biological processes, and molecular functions involved in defense responses than GPF DIA alone, defined as gene ontology analysis of samples obtained during Pierce’s disease symptom development (Table S8). Proteins with significant differential abundance in the diseased group were analyzed separately to detect enriched pathways under each condition (Figure 9 and Figure 10). Forty increased proteins were identified by GPF DIA and 74 by DIA+Prosit (Figure 9, Table S9) and a total of 41 decreased with GPF DIA and 62 with DIA+Prosit (Figure 10, Table S10) based on *Arabidopsis* best match ID.

Most enriched pathways identified using GPF DIA datasets were also present in DIA+Prosit. However, because DIA+Prosit identified more proteins, more significantly affected pathways were revealed. According to gene ontology analysis, proteins identified by GPF DIA that increased in diseased samples are involved in innate immune response, drug catabolic processes, aminoglycan catabolic process, activation of innate immune response, cell wall organization or biogenesis, MAPK signaling pathway, and response to cadmium ion. The pathways aminoglycan catabolic process and cell wall organization or biogenesis increased in both GPF DIA and DIA+Prosit data. The latter also revealed response to metal ion, immune response, response to bacterium, cofactor metabolic process, detoxification, reactive oxygen species metabolic process and defense response biological processes as significantly enriched.

The analysis of the decreased proteins in the diseased plants showed that except for the arabinan catabolic process, all identified pathways were significantly enriched in the DIA+Prosit approach, which listed also galactose metabolism, defense response signaling pathway and cell wall organization or biogenesis.

## 4. Discussion

This study illustrates the use of deep learning peptide predictions with data independent acquisition to identify the effects of Pierce’s disease in grapevine sap. Our data show the remarkable power of this approach to augment the molecular description of complex biological samples, exemplified here by the vascular sap of grapevines with Pierce’s disease. We used a pressure chamber to extract vascular leaf sap from grapevines comparing healthy and diseased plants and performed comparative proteome analysis. Previous studies of the grapevine xylem proteome provided important clues regarding the plant responses to infection; however, they also faced technical challenges in extracting enough material to describe the complexity of this pathosystem adequately. The vascular system is particularly crucial to understand Pierce’s disease as *Xf* cells are restricted to this microenvironment within plants. Thus, much of its interaction with the host occurs on the surface of xylem cells. As proteomic methods and equipment are evolving rapidly, we investigated the effect of a new deep neural proteome prediction method, Prosit, to increase identified proteins of data independent acquisition (DIA) mass spectrometry data. Our results suggest that incorporating a deep-learning architecture approach like Prosit with DIA data could help identify more protein candidates in response to pathogenesis and other biological phenomena. Prosit significantly increased the number of proteins, especially those in low abundance proteins, detected from both *Vitis* and *Xylella*, providing a more detailed picture of this plant–pathogen interaction.

### 4.1. A New Proteomic Approach for Vascular Sap Studies

Applying Prosit to the DIA data increased the number of detected proteins from 145 to 360 for grapevines, and from 18 to 90 proteins for *X. fastidiosa* compared to GPF DIA. Proteomics studies from vascular plant sap always face technical challenges because of the low protein concentration present in this plant organ. Previous studies identified differently expressed transcripts and proteins in grapevines using 2D-PAGE for protein isolation and further detection by MS/MS. The proteome depth for these sample types was ~100 proteins with molecular weights from 20 to 75 kDa, with >40 kDa [22]. The most recent proteomic study related to Pierce’s disease detected 91 proteins by LC-MS/MS that ranged from 12 to 114 kDa. That study incorporated structural information into the proteomic data analysis using CHURNER [26]. The number of identified peptides from these 91 proteins ranged from two to 23 peptides. In addition to identifying more proteins, DIA+Prosit also increased the number of peptides identified for each protein. This is significant because we required a minimum of two mapped peptides per protein for identification to increase the confidence and reduce protein false discoveries. The richer DIA+Prosit results provided a deeper comprehension of the relevant processes taking place during infection and the molecular functions that could be targeted with priority for increasing plant defense.

By combining DIA and Prosit, both the number of proteins and the sensitivity of detection increased. The proteins at low abundance were increased, and the accurate fragment intensity predictions from Prosit improved the quality of peptide identification in data searching [31]. The molecular weight of identified proteins in our study ranged from eight to 217 kDa, significantly broader than in previous studies. The smallest protein detected by DIA+Prosit was 8 kDa BBE domain-containing protein (VIT_10s0003g05430) observed by DIA+Prosit analysis with six exclusive peptides. This protein has been described previously as necessary in the plant–pathogen interaction of *Vitis* and *Botrytis cinerea*. Berberine bridge (BBE)-like enzymes inactivate oligo galacturonides (OGs), which accumulated as a result of polygalacturnonase-inhibiting protein (PGIP) activity induced by the infection process [47,48]. Oxidized OGs become less active as defense inducers and are less susceptible to hydrolysis by the pathogen’s polygalacturnonases (PGs). The accumulation of OGs can compromise plant growth and resistance through cell death induction. Therefore, the decrease in BBE-like enzymes in grapevines infected with *Xf* suggest a contribution to the plant’s susceptibility. This is the first detection of this protein in grape xylem sap, only achieved when combining DIA and Prosit.

The largest, 217 kDa protein was a member of the subtilase family (VIT_16s0098g00970), detected by both GPF DIA and DIA+Prosit. These proteins control establishment of systemic-induced resistance and immune priming by the detection of the biotic stimulus [49]. This protein was not detected in healthy plants in the GPF DIA data; only in the diseased plants with seven identified peptides. In the DIA+Prosit data, the number of observed peptides increased to nine and the protein was also detected in healthy samples, but at lower levels compared to the diseased (consistent with the GPF DIA results). This result exemplifies the increase in sensitivity by implementing Prosit in combination with DIA.

### 4.2. Plant Response to X. fastidiosa Infection as Assessed Using the Vascular Sap

Although the number of studies investigating expressed transcripts and proteins in the xylem sap of plants infected with *Xf* is small, they have provided valuable information regarding plant responses to infection [25,26,27,50]. By accurately evaluating the vascular leaf sap of plants infected with *Xf* using a more sensitive and reproducible proteomic approach, our study confirmed the presence of secreted proteins associated with pathogenesis-related (PR) proteins, chitinases, and β-1-3-glucanases as the key players in mediating the defense response upon pathogen infection [26]. Our study revealed β-1-3-glucanase 3 (VIT_08s0007g06060) as a vital protein contributing to the variance between healthy and diseased plants in the DIA+Prosit data (VIP score = 3.1) and the fourth most important in the GPF DIA data (VIP score = 2.7). Five β-1-3-glucanases were also detected in the DIA+Prosit data, but only four in the GPF DIA data. Except for one β-1-3-glucanase protein (VIT_06s0061g00100) that was not significantly decreased, all other β-1-3-glucanases in both data sets increased. β-1-3-glucanases belong to the PR2 class; their expression is induced by several pathogens including fungi, oomycetes and most recently discovered in bacterial infection [25,48,51,52]. Other PR proteins (including PR1), proteases, chitinases, and peroxidases were also confirmed in our study but in a greater number of proteins (Table 2). Chakraborty et al. (2016) detected 15 peroxidases, but our study increased this number to 20. This increase could be due to the Prosit predictions being generalized to non-tryptic peptides increasing peptide predictions [31] and represents yet another critical improvement as protein families with multiple members represented in a dataset gain higher scores in functional analyses such as gene ontology or pathway mapping.

## 5. Conclusions

This study is a successful example of using the GPF DIA approach combined with deep-learning neural network Prosit. Three hundred sixty proteins were identified and quantified from the xylem sap of grapevines subjected to *Xf* inoculation. We also identified different sets of proteins with altered abundance upon infection that were identified in previous proteomic studies and highlighted new, previously undetected low-molecular weight and low-abundance proteins. Prosit + DIA is especially useful in samples with lower protein abundance and diversity, providing more functional clues of significant players in proteome studies. The data analysis pipeline including Prosit and the necessary spectral libraries for both *V. vinifera* and *X. fastidiosa* are now available for future proteomic studies.

## Figures and Tables

**Table 1:** Overview of proteomics studies of grapevine vascular sap.

*Vitis sp.* Variety	Biological Material	*Xf* Inoc.	Method	Peptide Spectra Analysis	Total Proteins	Molecular Weight (kDa)	Matched Peptides	Signal Peptide	Ref.
Chardonnay	Xylem sap	No	2D-PAGE MALDI-TOF MS/MS	GPM	10	25–150	1	No	[21]
PD tolerant/susceptible varieties	Xylem sap	No	2D-PAGE LC-MS/MS	Mascot	100 *	20–75	1–4	No	[22]
PD tolerant/susceptible varieties	Stem	Yes	2D-PAGE nano-LC-MS/MS	Bioworks	200 *	14.4–45	2–32	No	[23]
Chardonnay	Leaf and apoplastic fluid	No	2D-PAGE MALDI-TOF MS/MS	Mascot	227	15–120	NA	No	[24]
PD tolerant/susceptible varieties	Xylem tissue	No	2D-PAGE MALDI-TOF MS/MS	Mascot	200 *	20–75	NA	No	[25]
Thompson Seedless	Xylem sap	Yes	LC-MS/MS	Scaffold	91	10–114	2–23	Yes	[26]
Thompson Seedless	Vascular leaf sap	Yes	LC-MS/MS	ScaffoldDIA (GPF)	145	12–217	2–22	Yes	This study
Thompson Seedless	Vascular leaf sap	Yes	LC-MS/MS	ScaffoldDIA+Prosit	360	8–217	2–31	Yes	This study

* Approximate number of total proteins; NA: not applicable.

**Table 2:** Pathogenesis-related (PR) proteins identified in the vascular leaf sap of grapevines showing Pierce’s disease symptoms by GPF DIA and DIA+Prosit with FDR < 0.01.

Accession Number	Arabidopsis Best Match	Protein Name	GPF DIA			DIA + Prosit		
			Matched Peptide	Ratio * (log2)	FDR	Matched Peptide	Ratio * (log2)	FDR
*Pathogenesis-related (PR1)*								
VIT_03s0088g00810	AT2G14610.1	pathogenesis-related gene 1	2	6.85	9.37 × 10^−12^	2	8.76	8.77 × 10^−13^
*Beta-1,3-glucanases (PR2)*								
VIT_08s0007g06060	AT3G57240.1	beta-1,3-glucanase 3	11	8.92	9.73 × 10^−17^	10	9.09	1.43 × 10^−9^
VIT_08s0007g06040	AT3G57240.1	beta-1,3-glucanase 3	9	5.36	8.31 × 10^−10^	6	5.50	2.82 × 10^−7^
VIT_06s0061g00120	AT3G57240.1	beta-1,3-glucanase 3	13	4.79	1.08 × 10^−10^	11	5.37	1.48 × 10^−9^
*Pathogenesis-related (PR3)*								
VIT_04s0008g00120	AT3G12500.1	basic chitinase	6	5.80	3.09 × 10^−13^	10	6.57	2.33 × 10^−12^
*Pathogenesis-related (PR4)*								
VIT_14s0081g00030	AT3G04720.1	pathogenesis-related 4	3	4.96	4.05 × 10^−11^	3	5.44	1.07 × 10^−8^
*Chitinases*								
VIT_16s0050g02230	AT5G24090.1	chitinase A	7	4.37	3.63 × 10^−4^	9	8.41	1.06 × 10^−12^
VIT_15s0046g01570	AT5G24090.1	chitinase A	3	3.54	1.36 × 10^−6^	3	3.44	3.72 × 10^−5^
VIT_11s0149g00380	AT4G19810.1	Glycosyl hydrolase **	5	−2.94	2.26 × 10^−4^	3	−2.47	9.29 × 10^−3^
VIT_11s0206g00030	AT4G19810.1	Glycosyl hydrolase **	4	−4.94	2.62 × 10^−7^	3	−4.44	1.60 × 10^−4^
VIT_16s0050g02210	AT5G24090.1	chitinase A				6	6.03	2.88 × 10^−8^
*Peroxidases*								
VIT_06s0004g07740	AT5G05340.1	Peroxidase superfamily	8	3.82	3.10 × 10^−7^	6	4.15	1.36 × 10^−6^
VIT_07s0191g00050	AT2G22420.1	Peroxidase superfamily	6	−3.53	1.46 × 10^−5^	7	−2.72	1.20 × 10^−3^
VIT_12s0055g01000	AT5G64120.1	Peroxidase superfamily	ND	ND	ND	7	−4.13	2.04 × 10^−3^

* Log_2_ ratio of diseased/healthy protein levels; ** family protein with chitinase insertion domain; ND: not detected.

## Supplementary Materials

The following are available online at https://www.mdpi.com/2079-7737/9/9/261/s1, Figure S1: Heatmaps for (a) GPF DIA and (b) DIA+Prosit; Figure S2: PLS-DA analysis of 139 proteins identified in both (a) GPF DIA and (b) DIA+Prosit methods and respective (c,d) cross-validation analysis; Table S1: Quantification of *Xf* cells by qPCR in leaf sap samples. Standard curve R^2^ = 0.985; Table S2: Proteomics data of *V. vinifera* leaf sap using data-independent acquisition (GPF DIA); Table S3: Proteomics data of *Xf* in the leaf sap using data-independent acquisition (GPF DIA); Table S4: Proteomics data of *V. vinifera* leaf sap using data-independent acquisition implemented with Prosit (DIA+Prosit) using edgeR package; Table S5: Proteomics data of *Xf* in the leaf sap using data-independent acquisition implemented with Prosit (DIA+Prosit); Table S6: Venn Diagram Analysis of *V. vinifera* proteome; Table S7: Venn Diagram Analysis of *Xf* proteome; Table S8: Gene ontology analysis of *V. vinifera* by Panther; Table S9: Summary of enrichment analysis by Metascape for increased proteins (*Arabidopsis* ID); Table S10: Summary of enrichment analysis by Metascape for decreased proteins (*Arabidopsis* ID), Notebooks (R): Gas-phase fractionation experimental library approach (DIA_Scaffold_edgeR.html), Prosit theoretical library approach (DIA_Prosit_edgeR.html).
