# Supplementary material for: Deep Learning Neural Network Prediction Method Improves Proteome Profiling of Vascular Sap of Grapevines during Pierce’s Disease Development
Source: Biology (Basel). 2020 Sep 1;9(9):261. doi: 10.3390/biology9090261 (PMC7565608; doi:10.3390/biology9090261)
Supplement: Supplementary file 1 [file biology-09-00261-s001.zip › Supplemental material final/DIA_Prosit_edgeR.html]

DIA\_Prosit\_edgeR\_withW3


# Analysis of Scaffold DIA experiment with edgeR¶

## Prosit theoretical library approach¶

### Deep learning neural network prediction method improves proteome profiling of vascular sap of grapevines during Pierce’s disease development¶

#### Notebook analysis performed by Phil Wilmarth, OHSU PSR Core, August 2020¶

## Overview and objectives¶

This is a study of grapevine vascular sap samples with and without Pierce's disease (a bacterial infection) using data independent acquisition (DIA). Two new approaches for identifying the peptides to be quantified were compared: using a gas phase fractionation approach to generate an experimental spectrum library for feature identification versus a theoretical library generated from the sequenced genomes using Prosit (one of several deep learning prediction tools).

The data were acquired on a Thermo Lumos Tribrid instrument with high-resolution parent and fragment ions. Both identification approaches were used with Scaffold DIA software.
The goal is to explore the differences in the data generated with the gas phase libraries versus the theoretical Prosit libraries. The data in this notebook is from the Prosit theoretical library approach.

The healthy and diseased samples were done at three time points (9 week, 12 week, and 15 week) in triplicate. The "W" samples are healthy and the "Y" samples are infected. One healthy sample at 12 weeks was lost during processing. Cluster analysis indicated that one of the 9 week samples (W3) was similar to the two 12 week samples (W5 and W6). This notebook uses W3, W5, and W6 in the "W" group to compare to the Y4, Y5, and Y6 samples.

More biological and experimental details can be found in the preprint.

## Differential expression (DE) testing¶

We will use the Bioconductor R package edgeR for the statistical testing. This widely used genomics tool has moderated test statistics and a robust trimmed mean of M-values normalization method.

> Robinson, M.D., McCarthy, D.J. and Smyth, G.K., 2010. edgeR: a Bioconductor package for differential expression analysis of digital gene expression data. Bioinformatics, 26(1), pp.139-140.
>
> Robinson, M.D. and Oshlack, A., 2010. A scaling normalization method for differential expression analysis of RNA-seq data. Genome biology, 11(3), p.R25.

---

## Load R libraries¶

In [1]:

```
# load libraries
library("tidyverse")
library("psych")
library("gridExtra")
library("scales")
library("limma") 
library("edgeR")
```

```
── Attaching packages ─────────────────────────────────────── tidyverse 1.3.0 ──

✔ ggplot2 3.3.0     ✔ purrr   0.3.3
✔ tibble  2.1.3     ✔ dplyr   0.8.5
✔ tidyr   1.0.2     ✔ stringr 1.4.0
✔ readr   1.3.1     ✔ forcats 0.5.0

── Conflicts ────────────────────────────────────────── tidyverse_conflicts() ──
✖ dplyr::filter() masks stats::filter()
✖ dplyr::lag()    masks stats::lag()


Attaching package: ‘psych’


The following objects are masked from ‘package:ggplot2’:

    %+%, alpha


Attaching package: ‘gridExtra’


The following object is masked from ‘package:dplyr’:

    combine


Attaching package: ‘scales’


The following objects are masked from ‘package:psych’:

    alpha, rescale


The following object is masked from ‘package:purrr’:

    discard


The following object is masked from ‘package:readr’:

    col_factor
```

## Define some common functions for the notebook¶

In [2]:

```
# ================== TMM normalization from DGEList object =====================
apply_tmm_factors <- function(y, color = NULL, plot = TRUE) {
    # computes the tmm normalized data from the DGEList object
        # y - DGEList object
        # returns a dataframe with normalized intensities
    
    # compute and print "Sample loading" normalization factors
    lib_facs <- mean(y$samples$lib.size) / y$samples$lib.size
    cat("\nLibrary size factors:\n", 
        sprintf("%-5s -> %f\n", colnames(y$counts), lib_facs))
    
    # compute and print TMM normalization factors
    tmm_facs <- 1/y$samples$norm.factors
    cat("\nTrimmed mean of M-values (TMM) factors:\n", 
        sprintf("%-5s -> %f\n", colnames(y$counts), tmm_facs))
    
    # compute and print the final correction factors
    norm_facs <- lib_facs * tmm_facs
    cat("\nCombined (lib size and TMM) normalization factors:\n", 
        sprintf("%-5s -> %f\n", colnames(y$counts), norm_facs))

    # compute the normalized data as a new data frame
    tmt_tmm <- as.data.frame(sweep(y$counts, 2, norm_facs, FUN = "*"))
    colnames(tmt_tmm) <- str_c(colnames(y$counts), "_tmm")
    
    # visualize results and return data frame
    if(plot == TRUE) {
        boxplot(log10(tmt_tmm), col = color, notch = TRUE, main = "TMM Normalized data")
    }
    tmt_tmm
}

# ================= reformat edgeR test results ================================
collect_results <- function(df, tt, x, xlab, y, ylab) {
    # Computes new columns and extracts some columns to make results frame
        # df - data in data.frame
        # tt - top tags table from edgeR test
        # x - columns for first condition
        # xlab - label for x
        # y - columns for second condition
        # ylab - label for y
        # returns a new dataframe
    
    # condition average vectors
    ave_x <- rowMeans(df[x])
    ave_y <- rowMeans(df[y])
    
    # FC, direction, candidates
    fc <- ifelse(ave_y > ave_x, (ave_y / ave_x), (-1 * ave_x / ave_y))
    direction <- ifelse(ave_y > ave_x, "up", "down")
    candidate <- cut(tt$FDR, breaks = c(-Inf, 0.01, 0.05, 0.10, 1.0), 
                     labels = c("high", "med", "low", "no"))
    
    # make data frame
    temp <- cbind(df[c(x, y)], data.frame(logFC = tt$logFC, FC = fc, 
                                          PValue = tt$PValue, FDR = tt$FDR, 
                                          ave_x = ave_x, ave_y = ave_y, 
                                          direction = direction, candidate = candidate, 
                                          Acc = tt$genes)) 
    
    # fix column headers for averages
    names(temp)[names(temp) %in% c("ave_x", "ave_y")]  <- str_c("ave_", c(xlab, ylab))    
    
    temp # return the data frame
}

# ============= log2 fold-change distributions =================================
log2FC_plots <- function(results, range, title) {
    # Makes faceted log2FC plots by candidate
        # results - results data frame
        # range - plus/minus log2 x-axis limits
        # title - plot title
    ggplot(results, aes(x = logFC, fill = candidate)) +
        geom_histogram(binwidth=0.1, color = "black") +
        facet_wrap(~candidate) +
        ggtitle(title) + 
        coord_cartesian(xlim = c(-range, range))
}

# ========== Setup for MA and volcano plots ====================================
transform <- function(results, x, y) {
    # Make data frame with some transformed columns
        # results - results data frame
        # x - columns for x condition
        # y - columns for y condition
        # return new data frame
    df <- data.frame(log10((results[x] + results[y])/2), 
                     log2(results[y] / results[x]), 
                     results$candidate,
                     -log10(results$FDR))
    colnames(df) <- c("A", "M", "candidate", "P")
    
    df # return the data frame
}

# ========== MA plots using ggplot =============================================
MA_plots <- function(results, x, y, title) {
    # makes MA-plot DE candidate ggplots
        # results - data frame with edgeR results and some condition average columns
        # x - string for x-axis column
        # y - string for y-axis column
        # title - title string to use in plots
        # returns a list of plots 
    
    # uses transformed data
    temp <- transform(results, x, y)
    
    # 2-fold change lines
    ma_lines <- list(geom_hline(yintercept = 0.0, color = "black"),
                     geom_hline(yintercept = 1.0, color = "black", linetype = "dotted"),
                     geom_hline(yintercept = -1.0, color = "black", linetype = "dotted"))

    # make main MA plot
    ma <- ggplot(temp, aes(x = A, y = M)) +
        geom_point(aes(color = candidate, shape = candidate)) +
        scale_y_continuous(paste0("logFC (", y, "/", x, ")")) +
        scale_x_continuous("Ave_intensity") +
        ggtitle(title) + 
        ma_lines
    
    # make separate MA plots
    ma_facet <- ggplot(temp, aes(x = A, y = M)) +
        geom_point(aes(color = candidate, shape = candidate)) +
        scale_y_continuous(paste0("log2 FC (", y, "/", x, ")")) +
        scale_x_continuous("log10 Ave_intensity") +
        ma_lines +
        facet_wrap(~ candidate) +
        ggtitle(str_c(title, " (separated)"))

    # make the plots visible
    print(ma)
    print(ma_facet)
}    

# ========== Scatter plots using ggplot ========================================
scatter_plots <- function(results, x, y, title) {
    # makes scatter-plot DE candidate ggplots
        # results - data frame with edgeR results and some condition average columns
        # x - string for x-axis column
        # y - string for y-axis column
        # title - title string to use in plots
        # returns a list of plots
    
    # 2-fold change lines
    scatter_lines <- list(geom_abline(intercept = 0.0, slope = 1.0, color = "black"),
                          geom_abline(intercept = 0.301, slope = 1.0, color = "black", linetype = "dotted"),
                          geom_abline(intercept = -0.301, slope = 1.0, color = "black", linetype = "dotted"),
                          scale_y_log10(),
                          scale_x_log10())

    # make main scatter plot
    scatter <- ggplot(results, aes_string(x, y)) +
        geom_point(aes(color = candidate, shape = candidate)) +
        ggtitle(title) + 
        scatter_lines

    # make separate scatter plots
    scatter_facet <- ggplot(results, aes_string(x, y)) +
        geom_point(aes(color = candidate, shape = candidate)) +
        scatter_lines +
        facet_wrap(~ candidate) +
        ggtitle(str_c(title, " (separated)")) 

    # make the plots visible
    print(scatter)
    print(scatter_facet)
}

# ========== Volcano plots using ggplot ========================================
volcano_plot <- function(results, x, y, title) {
    # makes a volcano plot
        # results - a data frame with edgeR results
        # x - string for the x-axis column
        # y - string for y-axis column
        # title - plot title string
    
    # uses transformed data
    temp <- transform(results, x, y)
    
    # build the plot
    ggplot(temp, aes(x = M, y = P)) +
        geom_point(aes(color = candidate, shape = candidate)) +
        xlab("log2 FC") +
        ylab("-log10 FDR") +
        ggtitle(str_c(title, " Volcano Plot"))
}

# ============== individual protein expression plots ===========================
# function to extract the identifier part of the accesssion
get_identifier <- function(accession) {
#    identifier <- str_split(accession, "\\|", simplify = TRUE)
#    identifier[,3]
    identifier <- accession
}

set_plot_dimensions <- function(width_choice, height_choice) {
    options(repr.plot.width=width_choice, repr.plot.height=height_choice)
}

plot_top_tags <- function(results, nleft, nright, top_tags) {
    # results should have data first, then test results (two condition summary table)
    # nleft, nright are number of data points in each condition
    # top_tags is number of up and number of down top DE candidates to plot
    # get top ipregulated
    up <- results %>% 
        filter(logFC >= 0) %>%
        arrange(FDR)
    up <- up[1:top_tags, ]
    
    # get top down regulated
    down <- results %>% 
        filter(logFC < 0) %>%
        arrange(FDR)
    down <- down[1:top_tags, ]
    
    # pack them
    proteins <- rbind(up, down)
        
    color = c(rep("red", nleft), rep("blue", nright))
    for (row_num in 1:nrow(proteins)) {
        row <- proteins[row_num, ]
        vec <- as.vector(unlist(row[1:(nleft + nright)]))
        names(vec) <- colnames(row[1:(nleft + nright)])
        title <- str_c(get_identifier(row$Acc), ", int: ", scientific(mean(vec), 2), 
                       ", FDR: ", scientific(row$FDR, digits = 3), 
                       ", FC: ", round(row$FC, digits = 1),
                       ", ", row$candidate)
        barplot(vec, col = color, main = title,
                cex.main = 1.0, cex.names = 0.7, cex.lab = 0.7)
    }    
}

# ============== p-value distribution =========================================
pvalue_plot <- function(results, title) {
    # Makes p-value distribution plots
        # results - results data frame
        # title - plot title
    ggplot(results, aes(PValue)) + 
        geom_histogram(bins = 100, fill = "white", color = "black") +
        geom_hline(yintercept = mean(hist(results$PValue, breaks = 100, 
                                     plot = FALSE)$counts[26:100]), na.rm = TRUE) +
        ggtitle(str_c(title, " p-value distribution"))
}
```

## Read in the prepped data¶

The original data contained a small proportion of missing values. The average intensity per protein across all samples was computed and proteins ranked by decreasing average intensity. Seven proteins had an average of less than 150,000 and were excluded. Those proteins had about 1/3 of all missing values.

For each biological sample, the smallest non-missing values were found. Based on the median value of the smallest values (about 9,000), missing values were replaced by a value of 1,500.

In [3]:

```
# read the protein-level quantitative values
dia_start <- read_tsv("PW-filtered_Prosit-DIA.txt")
```

```
Parsed with column specification:
cols(
  Accession = col_character(),
  W1 = col_double(),
  W2 = col_double(),
  W3 = col_double(),
  W6 = col_double(),
  W5 = col_double(),
  W8 = col_double(),
  W7 = col_double(),
  Y1 = col_double(),
  Y3 = col_double(),
  Y2 = col_double(),
  Y5 = col_double(),
  Y4 = col_double(),
  Y6 = col_double()
)
```

In [4]:

```
# extract protein accession column and the actual data
# separate accessions from the data
accessions <- dia_start$Accession
dia_data <- dia_start %>% select(-Accession)

head(dia_data)
length(accessions)
```

A tibble: 6 × 13

| W1 | W2 | W3 | W6 | W5 | W8 | W7 | Y1 | Y3 | Y2 | Y5 | Y4 | Y6 |
| --- | --- | --- | --- | --- | --- | --- | --- | --- | --- | --- | --- | --- |
| <dbl> | <dbl> | <dbl> | <dbl> | <dbl> | <dbl> | <dbl> | <dbl> | <dbl> | <dbl> | <dbl> | <dbl> | <dbl> |
| 1445439771 | 9549925860 | 616595002 | 389045145 | 1000000000 | 2398832919 | 3630780548 | 3981071706 | 2137962090 | 4570881896 | 15848931925 | 10000000000 | 6760829754 |
| 2089296131 | 2630267992 | 5495408739 | 2951209227 | 4466835922 | 2818382931 | 2818382931 | 1318256739 | 2454708916 | 2137962090 | 3467368505 | 3388441561 | 2344228815 |
| 501187234 | 3715352291 | 416869383 | 208929613 | 457088190 | 501187234 | 630957344 | 1819700859 | 2884031503 | 3162277660 | 9772372210 | 4365158322 | 3981071706 |
| 588843655 | 1659586907 | 1174897555 | 177827941 | 1023292992 | 645654229 | 724435960 | 870963590 | 707945784 | 1445439771 | 1258925412 | 2290867653 | 1318256739 |
| 34673685 | 2137962090 | 24547089 | 33884416 | 56234133 | 102329299 | 125892541 | 467735141 | 407380278 | 794328235 | 5011872336 | 2511886432 | 1737800829 |
| 851138038 | 1288249552 | 933254301 | 501187234 | 1202264435 | 977237221 | 891250938 | 758577575 | 562341325 | 912010839 | 1513561248 | 1318256739 | 891250938 |

353

### Load the data into an `DGEList` edgeR object¶

In [5]:

```
# define indices for conditions of interest
W_start <- c(3, 5, 4) # W3, W5, W6 - healthy group
Y_start <- c(12, 11, 13) # Y4, Y5, Y6 - infected group

# load data into DGEList object
group <- c(rep("W", 3), rep("Y", 3))
y <- DGEList(counts = dia_data[c(W_start, Y_start)], group = group, genes = accessions)
y$samples
```

A data.frame: 6 × 3

|  | group | lib.size | norm.factors |
| --- | --- | --- | --- |
|  | <fct> | <dbl> | <dbl> |
| W3 | W | 29435480911 | 1 |
| W5 | W | 28363818617 | 1 |
| W6 | W | 20329047105 | 1 |
| Y4 | Y | 52607210721 | 1 |
| Y5 | Y | 68060530231 | 1 |
| Y6 | Y | 42940332686 | 1 |

### Normalize the TMT data¶

EdgeR normalization is actually done in two steps. The first, called a library size adjustment, is like a sample loading normalization. This gets rid of the big differences between samples so that the TMM algorithm has better starting data. We will need to compute the normalized intensities from the TMM factors (edgeR internally uses the factors).

In [6]:

```
# run the TMM normalization
y <- calcNormFactors(y)

# set colors for plotting
colors <- c(rep("red", 3), rep("blue", 3))

# redefine the indices for the subsetted data
W <- 1:3
Y <- 4:6

# get the normalized data values
dia_tmm <- apply_tmm_factors(y, colors)

# check the clustering
plotMDS(y, col = colors, main = "Samples after TMM")
```

```
Library size factors:
 W3    -> 1.368736
 W5    -> 1.420451
 W6    -> 1.981864
 Y4    -> 0.765853
 Y5    -> 0.591964
 Y6    -> 0.938265

Trimmed mean of M-values (TMM) factors:
 W3    -> 0.707656
 W5    -> 0.598606
 W6    -> 0.557908
 Y4    -> 1.500733
 Y5    -> 2.169105
 Y6    -> 1.299842

Combined (lib size and TMM) normalization factors:
 W3    -> 0.968594
 W5    -> 0.850290
 W6    -> 1.105697
 Y4    -> 1.149341
 Y5    -> 1.284033
 Y6    -> 1.219596
```

### Compute dispersion for edgeR modeling¶

edgeR uses trended dispersion to moderate the testing statistics to make the modeling more robust for studies with small replicate numbers.

In [7]:

```
# we need to get dispersion estimates
y <- estimateDisp(y)
plotBCV(y, main = "Dispersion trends")
```

```
Design matrix not provided. Switch to the classic mode.
```

### edgeR exact test¶

We will use the exact test in edgeR for this simple two-state comparison. We will also simplify/reformat the test results and save them in a data frame.

In [8]:

```
# compute the exact test models, p-values, FC, etc.
et <- exactTest(y, pair = c("W", "Y"))

# check some top tags
topTags(et)$table

# this counts up, down, and unchanged genes (proteins) at 5% FDR
summary(decideTestsDGE(et, p.value = 0.05))

# make the results table 
tt <- topTags(et, n = Inf, sort.by = "none")$table
exact <- collect_results(dia_tmm, tt, W, "W", Y, "Y")

# make an MD plot (like MA plot)
plotMD(et, p.value = 0.05)
abline(h = c(-1, 1), col = "black")
```

A data.frame: 10 × 5

|  | genes | logFC | logCPM | PValue | FDR |
| --- | --- | --- | --- | --- | --- |
|  | <chr> | <dbl> | <dbl> | <dbl> | <dbl> |
| 5 | VIT\_03s0091g00160.t01 | 6.713087 | 15.544210 | 4.299892e-18 | 1.517862e-15 |
| 13 | VIT\_02s0025g04330.t01 | 5.713937 | 14.287486 | 3.492991e-17 | 6.165129e-15 |
| 104 | VIT\_04s0008g00140.t01 | 6.855611 | 10.556781 | 2.821234e-16 | 3.319652e-14 |
| 290 | VIT\_07s0005g00720.t01 | -10.617880 | 5.555447 | 4.685949e-15 | 4.135350e-13 |
| 33 | VIT\_03s0088g00810.t01 | 8.759101 | 13.624736 | 1.242211e-14 | 8.770008e-13 |
| 86 | VIT\_14s0030g01890.t01 | 5.841720 | 10.925355 | 1.747453e-14 | 1.028085e-12 |
| 53 | VIT\_16s0050g02230.t01 | 8.412190 | 11.992133 | 2.110932e-14 | 1.064513e-12 |
| 14 | VIT\_04s0008g00120.t01 | 6.568862 | 14.738642 | 5.272745e-14 | 2.326599e-12 |
| 56 | VIT\_18s0001g11180.t01 | 9.158573 | 12.167621 | 5.181224e-13 | 2.032191e-11 |
| 111 | VIT\_07s0005g00730.t01 | -4.929421 | 9.952323 | 6.703464e-12 | 2.366323e-10 |

```
       Y-W
Down    65
NotSig 204
Up      84
```

### Check if testing looks okay¶

It is important to see if the modeling looks reasonable. Our general assumptions are that we have a large fraction of the proteins that are not differentially expressed. Those will have a uniform (flat) p-value distribution from 0.0 to 1.0. We also expect (hopefully) some true differential expression candidates. Those should have very small p-values and have a sharper distribution at low p-values.

In [9]:

```
# check the p-value distrubution
pvalue_plot(exact, "W vs Y, Scaffold")
```

### We have a rather sparse distribution¶

Despite the sparse data, we still observe the two distributions of p-values, so the testing seems reasonable.

### We can categorize candidates by ranges of adjusted p-values¶

We can define three cuts on the FDR: 10% to 5% are "low" significance, 5% to 1% are medium significance, and less than 1% are more "highly" significant. Cut values can be adjusted depending on the experimental situation. We can look at expression ratio distributions as a function of candidate category. If variance is not too variable protein-to-protein, then we would expect larger mean differences to be associated with lower FDR values. Faceted plotting in ggplot2 is another way to see patterns in data.

In [10]:

```
# see how many candidates are in each category
exact %>% count(candidate)

# can look at log2FC distributions as a check
log2FC_plots(exact, 4, "LogFC by candidate for W vs Y")
```

A tibble: 4 × 2

| candidate | n |
| --- | --- |
| <fct> | <int> |
| high | 109 |
| med | 40 |
| low | 32 |
| no | 172 |

### Visualize the edgeR DE candidates¶

- MA plots
- scatter plots
- volcano plot

We need some transformed axes for MA plots and for volcano plots. We will make a function for that and also some functions for the plotting. MA plots first. The dotted lines indicate 2-fold changes.

In [11]:

```
# MA plots of DE candidates
MA_plots(exact, "ave_W", "ave_Y", "W versus Y")
```

### Scatter plots¶

The solid diagonal line is 1:1, the dotted lines are 2-fold changes. The axes are in log scale.

In [12]:

```
# scatter plots
scatter_plots(exact, "ave_W", "ave_Y", "W versus Y")
```

### Volcano plot¶

Volcano plots are another common way to visualize DE candidates.

In [13]:

```
# finally, a volcano plot
volcano_plot(exact, "ave_W", "ave_Y", "W versus Y")
```

### Plot the intensities for some of the top DE candidates by FDR¶

We can see how the intensities of the individual samples compare for the top 10 up- and down-regulated DE candidate proteins.

In [14]:

```
# plot the top 10 up and 10 down proteins
set_plot_dimensions(7, 4)
plot_top_tags(exact, 3, 3, 10)
set_plot_dimensions(7, 7)
```

## Summary¶

We had many more quantified proteins with the Prosit approach and there are clearly many significant expression differences. The normalizations, testing results, and individual protein expression levels all fit together and support large-scale changes between healthy and diseased grape sap.

We should always end notebooks with information about what packages and versions were used in the analysis.

In [15]:

```
# save the testing results
write.table(exact, file = "DIA-Prosit_results_W3.txt", sep = "\t",
            row.names = FALSE, na = " ")

# log the session
sessionInfo()
```

```
R version 3.5.3 (2019-03-11)
Platform: x86_64-apple-darwin15.6.0 (64-bit)
Running under: macOS  10.15.6

Matrix products: default
BLAS: /Library/Frameworks/R.framework/Versions/3.5/Resources/lib/libRblas.0.dylib
LAPACK: /Library/Frameworks/R.framework/Versions/3.5/Resources/lib/libRlapack.dylib

locale:
[1] en_US.UTF-8/en_US.UTF-8/en_US.UTF-8/C/en_US.UTF-8/en_US.UTF-8

attached base packages:
[1] stats     graphics  grDevices utils     datasets  methods   base     

other attached packages:
 [1] edgeR_3.24.3    limma_3.38.3    scales_1.1.0    gridExtra_2.3  
 [5] psych_1.9.12.31 forcats_0.5.0   stringr_1.4.0   dplyr_0.8.5    
 [9] purrr_0.3.3     readr_1.3.1     tidyr_1.0.2     tibble_2.1.3   
[13] ggplot2_3.3.0   tidyverse_1.3.0

loaded via a namespace (and not attached):
 [1] Rcpp_1.0.4       locfit_1.5-9.1   lubridate_1.7.4  lattice_0.20-40 
 [5] assertthat_0.2.1 digest_0.6.25    IRdisplay_0.7.0  R6_2.4.1        
 [9] cellranger_1.1.0 repr_1.1.0       backports_1.1.5  reprex_0.3.0    
[13] evaluate_0.14    httr_1.4.1       pillar_1.4.3     rlang_0.4.5     
[17] uuid_0.1-4       readxl_1.3.1     rstudioapi_0.11  labeling_0.3    
[21] splines_3.5.3    munsell_0.5.0    broom_0.5.2      compiler_3.5.3  
[25] modelr_0.1.6     pkgconfig_2.0.3  base64enc_0.1-3  mnormt_1.5-6    
[29] htmltools_0.4.0  tidyselect_1.0.0 fansi_0.4.1      crayon_1.3.4    
[33] dbplyr_1.4.2     withr_2.2.0      grid_3.5.3       nlme_3.1-145    
[37] jsonlite_1.6.1   gtable_0.3.0     lifecycle_0.2.0  DBI_1.1.0       
[41] magrittr_1.5     cli_2.0.2        stringi_1.4.6    farver_2.0.3    
[45] fs_1.3.2         xml2_1.2.5       generics_0.0.2   vctrs_0.2.4     
[49] IRkernel_1.1     tools_3.5.3      glue_1.3.2       hms_0.5.3       
[53] parallel_3.5.3   colorspace_1.4-1 rvest_0.3.5      pbdZMQ_0.3-3    
[57] haven_2.2.0
```

In [ ]:

```

```
