# Supplementary figures and images for: Deep Learning Neural Network Prediction Method Improves Proteome Profiling of Vascular Sap of Grapevines during Pierce’s Disease Development
Source: Biology (Basel). 2020 Sep 1;9(9):261. doi: 10.3390/biology9090261 (PMC7565608; doi:10.3390/biology9090261)

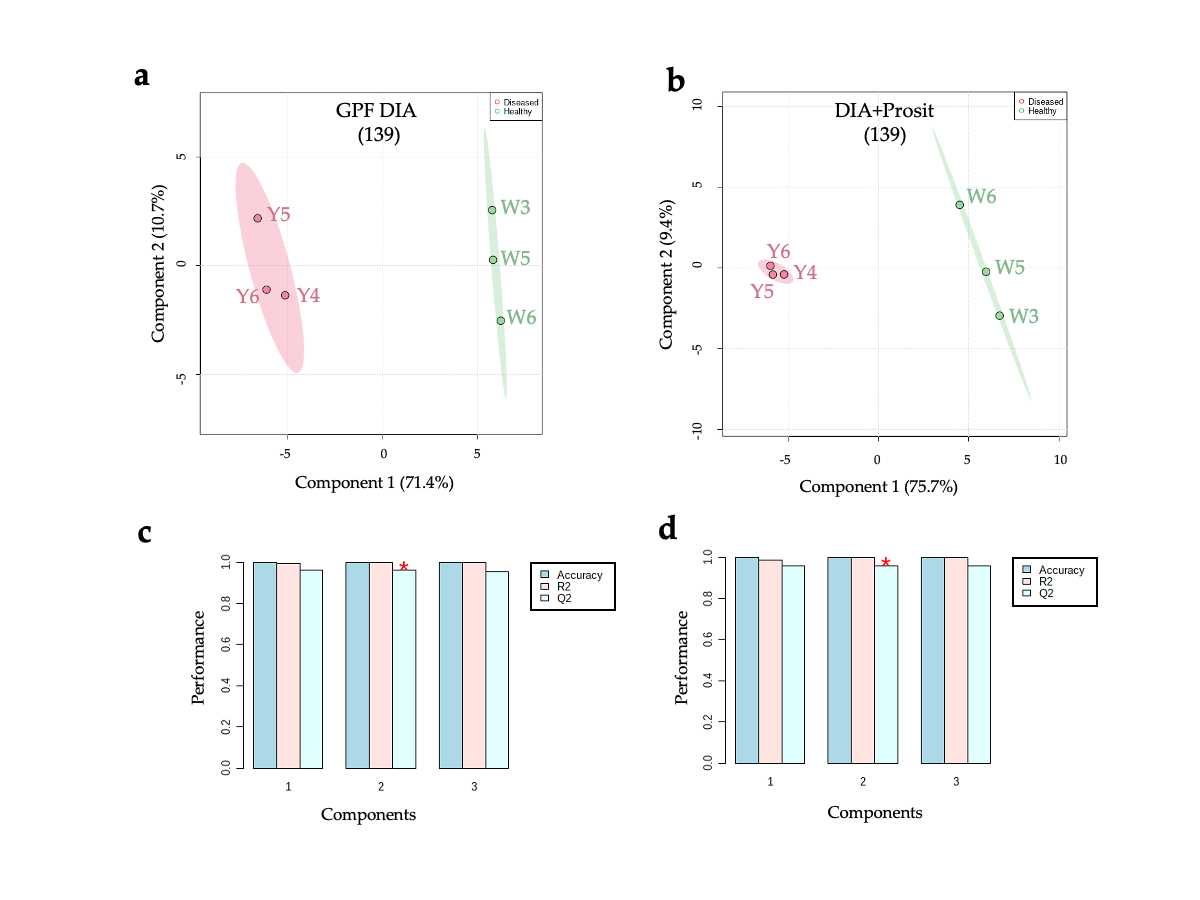

Supplement: Supplementary file 1 [file biology-09-00261-s001.zip › Supplemental material final/Figure S2_v.2.png]

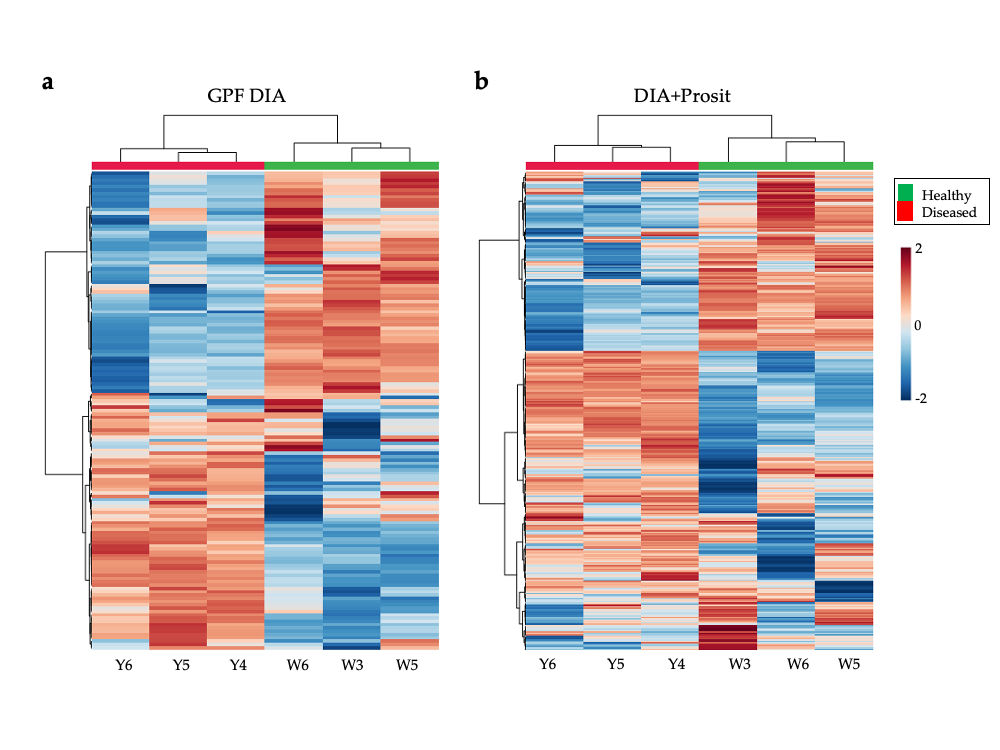

Supplement: Supplementary file 1 [file biology-09-00261-s001.zip › Supplemental material final/Figure S1.png]
